# Supplementary material for: Mapping-free variant calling using haplotype reconstruction from k-mer frequencies
Source: Bioinformatics. 2017 Nov 24;34(10):1659–65. doi: 10.1093/bioinformatics/btx753 (PMC5946877; doi:10.1093/bioinformatics/btx753)
Supplement: Supplementary Data [file btx753_kestrelsupplementary.pdf]

Mapping-free variant calling using haplotype reconstruction from k-mer frequencies
Supplementary material

Peter Audano, Shashidhar Ravishankar, and Fredrik Vannberg

Contents

1 Kestrel algorithm definition **2**
1.1 Overview . . . 2
1.1.1 Sequence k-merization . . . 2
1.1.2 Active region detection . . . 3
1.1.3 Local assembly over active regions . . . 3
1.1.4 Variant calling . . . 3
1.2 Preliminaries . . . 3
1.3 Locating active regions . . . 4
1.4 Alignment parameters . . . 5
1.5 Alignment data structures . . . 5
1.6 Alignment score matrices . . . 6
1.7 Alignment extension . . . 7
1.8 Maximum score and optimal alignments . . . 7
1.9 Alignment termination . . . 7
1.10 Haplotypes . . . 8
1.11 Parameter selection . . . 8

2 Kestrel implementation **9**
2.1 Alignment matrices . . . 9
2.2 Active region heuristics . . . 10
2.2.1 Exponential decay . . . 10
2.2.2 Peak detection . . . 11

3 *S. pneumoniae* test case **12**
3.1 Sequence data . . . 12
3.2 Variant calling . . . 12
3.2.1 Assembly approach and variant verification . . . 12
3.2.2 Alignment approach . . . 13
3.2.3 Kestrel approach . . . 13
3.3 Comparing variants . . . 14
3.4 Removed samples . . . 15
3.5 Results . . . 15
3.6 Data storage . . . 17
3.7 Variant Call Depth . . . 17

4 *E. coli* test case **19**

5 Automated test cases **19**
5.1 *S. pneumoniae* . . . 20

5.2 *N. meningitidis* 21
5.3 *E. coli* 21

1 Kestrel algorithm definition

The input of the algorithm is short-read sequence data and one or more reference sequences. The output is a set of variant calls.

Kestrel is both a tool and a proof-of-concept. It is a tool that works well in the absence of low-complexity or repetitive regions. Its application to most genomes should be done with care. It cannot resolve paralogues, and it is therefore subject to false-positive calls due to paralogue-specific variants (PSVs). Kestrel does variant calling on regions, and if a variant region is too large, it's memory consumption can be prohibitive. These missed regions lead to false-negatives. It can, however, operate in regions where the reference does not represent the sample and therefore causes sequence read alignments to fail.

As a proof of concept, Kestrel is a purely k-mer based approach to variant calling. Modern k-mer methods are limited to SNP calling in limited contexts[1,2], but Kestrel can resolve variant regions much larger than the k-mers themselves. Other methods require mapping sequence reads to the reference, assembling into contigs, or building de Bruijn graphs. Kestrel performs a k-mer based local assembly guided by a novel alignment algorithm. While it is not technically alignment-free, it does not require mapping sequence reads to the reference or assembling all sequence reads or k-mers. It does these steps in a more targeted fashion. In some contexts, this leads to a very fast variant calling algorithm that can work in regions where alignments fail without needing a whole genome assembly or de Bruijn graphs. Applied to whole genomes, however, Kestrel has its limitations. The body of the main publication outlines these advantages and disadvantages in more detail.

This section defines the algorithm used by Kestrel to identify variants. Like many rigorous definitions, implementing it directly as shown would not yield an optimized solution. **Supplementary Section 2** discusses how this specification was transformed into a functioning software application.

1.1 Overview

Kestrel has several key parts:

1. Sequence k-merization
2. Active region detection
3. Local assembly over active regions
4. Variant calling

1.1.1 Sequence k-merization

All sequence reads are first transformed into k-mers. The first k-mer of a read is the first k bases. The second k-mer is the k bases starting with the second base in the read. The remaining k-mers are found by moving one base at a time until the end of the k-mer reaches the last base of the sequence. Any k-mers containing an ambiguous base, such as N, are discarded. Each unique k-mer is counted and stored in a file where the counts can be rapidly queried using the k-mer as a key. In the Kestrel implementation, this process is carried out by the KAnalyze[3] API.

The reference sequence is also transformed to k-mers, but these are left in order and not counted like the sequence reads.

1.1.2 Active region detection

Active region detection starts by taking the reference k-mers, and for each reference k-mer, assigning the count for that k-mer from the sequence data. Given a complex reference region free of repeats and paralogues, the distribution of k-mer counts over the reference k-mers will be roughly uniform.

Any variant (SNP, insertion, or deletion) changes k-mers in the reference. Therefore, the k-mer counts should drop abruptly for some number of k-mers and return to the same uniform distribution some number of k-mers downstream. For example, a SNP changes k consecutive k-mers to 0. Insertions change $k - 1$ k-mers at the reference breakpoint, and deletions change $k + n - 1$ k-mers, where n is the length of the deletion. Real data is not this clean, but the specification below outlines how this is managed.

The active region is all bases with a low-frequency k-mer plus the bases covering the high frequency k-mers on either end. These high-frequency k-mers serve as anchors for local assembly.

When a variant region is close to the left or right end of a reference sequence, then an active region may be defined that has no anchor k-mer on that end. In this case, variant recovery starts from the single anchor k-mer and continues to the end. This document focuses on the case where the anchor k-mer is on the left and the assembly extends from left to right. However, the same algorithm can be applied in reverse for the case where there is one anchor k-mer on the right and none on the left.

1.1.3 Local assembly over active regions

An anchor k-mer can be shifted one base upstream by removing the first base on the left and appending a base on the right. The k-mer is moved one base right, but to a computer scientist, this is achieved by left-shifting the k-mer and masking off the left-most base. Each of the four bases (A, C, G, and T) are inserted into the right-most position of this shifted k-mer in turn. The base that recovers a k-mer with a high count from the sequence k-mers reveals the next base in the sequence. This process is repeated for the new k-mer until the end of the active region is reached.

This process is guided by a modified Smith-Waterman algorithm that determines when to stop the local assembly. It is designed so the maximum alignment score is achieved when the assembly reaches the anchor k-mer on the other end. This algorithm also puts a limit on how much time and memory is used on erroneous alignment branches. Like any other affine-gap model, this algorithm can be tuned to determine how large variant regions may be before terminating them. Although careful memory-management techniques have been implemented, memory consumption on large active regions can still be very high.

1.1.4 Variant calling

The start position of the alignment is known from active region detection, and the alignment events are known from the alignment algorithm. Tracing the alignment back gives each variant. A mismatched base is a SNP, a gap in the reference in an insertion, and a gap in the assembled sequence is a deletion.

1.2 Preliminaries

Given a known reference sequence, X, identify regions, x, which are suspected to contain variants, and dynamically find one or more haplotypes, y, that align with x using evidence from sequence reads. x is referred to as an *active region*, and each y as a *haplotype* sequence over x. x and y align end-to-end, but every base may not match. Building y and calling variants from it is the aim of the Kestrel algorithm. The terms "active region" and "haplotype" are borrowed from the GATK[4] HaplotypeCaller.

This algorithm definition will illustrate the how k-mer frequencies are employed to identify active regions, x, and to choose bases for building the halpotype, y. For each read in a set of sequence data for a given sample, all substrings of length k are extracted. Each of these substrings is called a k-mer.

The k-mer frequency is defined as the number of times each k-mer was observed in the sequence data. This k-mer counting problem has been solved several times, and several software implementations are available[3,5,6]. Here, we use KAnalyze[3] because we have built features into it that support efficient k-mer frequency querying as well as arbitrarily large k-mers.

X := Full reference string.
x := A substring of X, called the active region, where variant detection occurs.
y := A haplotype over x, which is discovered by the Kestrel algorithm.
k := The number of bases in each k-mer.

1.3 Locating active regions

N is an array of frequencies for each k-mer in the reference sequence, X, from the first k-mer (with frequency N_1) on the left end to the last k-mer on the right end of X. The frequency of each k-mer in N is the count of that k-mer in the sample sequence data. $N_i = 0$ if the k-mer at position i contains ambiguous bases. If X contains no variants, and therefore no active regions, the distribution of the frequencies in N is roughly uniform with some fluctuation due to sequence read errors, non-uniform read coverage, k-mer overlap with other regions of the genome, and other sequencing anomalies.

N is traversed from left to right searching for a sharp decline or increase of the frequency between neighboring k-mers N_i and N_{i+1}, which may indicate the edge of an active region. The threshold, ϵ, is the magnitude of difference between N_i and N_{i+1} that must be exceeded to trigger an active region scan.

If $N_i > N_{i+1} + \epsilon$, then the active regions spans from N_i to some downstream k-mer, $i + l$, where the k-mer frequency returns to a value near N_i. The active region, x, defined by this range includes both N_i and N_{i+l}, which have a high frequency compared to the rest of the k-mers between them. These end k-mers are called the *anchor k-mers* of the active region as they help seed and terminate the process of building y. If y does not start and end with these anchor k-mers, then y is rejected.

The shortest active region is the case when one or more bases are inserted and no other variants occur within k bases of the insertion. In this case, the number of k-mers with a frequency affected by this insertion will be $k - 1$. Since an active region, x, includes one unaltered k-mer on each end, x must span $k + 1$ k-mers or it is rejected.

If a variant occurs less than k bases from the right end of the sequence, X, then there will be no anchor k-mer on the right side because all k-mers up to the end of X are altered. In this case, x may be left un-anchored on its right end, and y is not required to match the missing anchor k-mer. y must also be allowed to end with a deletion from x. Since the evidence for discovered variants is not as strong, Kestrel requires both anchor k-mers by default.

If $N_i < N_{i+1} - \epsilon$, then the k-mer frequencies increased significantly, and an active region may be present from N_1 to N_{i+1}. Similar to an active region scan that reaches the right end of N, this will occur if variants are found less than k bases from the left end of X. This region is anchored on the right side by N_{i+1}, but it is not anchored on the left side. In this case, y may begin with a deletion on the left side. As with the case where the right anchor k-mer is missing, this active region will be ignored by default because the evidence for variants will not be as strong.

This section ignores several details, such as how to choose ϵ or deal with noisy data. These issues will be addressed in **Supplementary Section 1.11**.

N := A set of k-mer frequencies ordered by the k-mers in X.
ϵ := K-mer frequency difference threshold.
l := The number of k-mers from the first low-frequency k-mer to the right anchor k-mer.

1.4 Alignment parameters

The alignment step is a dynamic programming algorithm based on the well known Smith-Waterman[7] algorithm. The key difference is that only the active region sequence, x, is known *a priori*, and each haplotype, y, is built using a local *de novo* assembly approach that terminates when an optimal alignment obtained. All acceptable alignments must match each base of the left anchor k-mer in x and y, but the right end of y is not known. Since this alignment must guide the process of building y, it must anchor on one side, but extend arbitrarily as y is reconstructed.

Two key modifications were made to Smith-Waterman; (i) any subalignment with a score of 0 cannot be extended, and (ii) the alignment must begin with a score greater than 0. These modifications force all possible alignments terminate on the left side as if it were a global alignment, but update dynamically as y is extended. **Supplementary Section 1.9** outlines how the scores are used to determine when to stop building y.

As with other alignment algorithms, a set of scoring criteria is required, and the alignment is optimized over these parameters. When two bases are aligned, R_{match} is added to the alignment score if the bases match, and $R_{mismatch}$ is added when the bases do not match. For each base aligned with a gap, R_{gap} is added to the score. R_{open} is added to the score for each time a gap is opened, i.e., once for every maximal substring consisting of gap character, "-". The initial score of the alignment is R_{init}, as required by our modification to Smith-Waterman. Note that R_{match} and R_{init} are strictly positive, $R_{mismatch}$, and R_{gap} are strictly negative, and R_{open} is non-positive. The optimal score matrix will be determined by the application, such as how divergent the sequences may be and the size of allowable insertion or deletion events.

$$\begin{aligned}
R_{match} &:= \text{Aligned bases match, } R_{match} > 0 \\
R_{mismatch} &:= \text{Aligned bases do not match, } R_{mismatch} < 0 \\
R_{open} &:= \text{A gap was opened, } R_{open} \leq 0 \\
R_{gap} &:= \text{Base aligned with a gap, } R_{gap} < 0 \\
R_{init} &:= \text{Initial alignment score, } R_{init} > 0
\end{aligned}$$

For convenience, we define a function, $match(i, j)$, to return the appropriate score for aligned bases, as shown by **Supplementary Eqn. 1**.

$$match(i,j) = \begin{cases} R_{match} & : x_i = y_j \\ R_{mismatch} & : x_i \neq y_j \end{cases} \tag{1}$$

1.5 Alignment data structures

The optimal alignment has the highest score of all possible alignments of x and y. Trying all possible alignments is clearly an inefficient way of solving the problem, however, the score of one particular alignment can be seen as the score of the same alignment one position shorter plus the score of the last position[8]. The dynamic programming solution *memoizes** the score of shorter alignments in a score matrix instead of recomputing it.

The alignment data structures are analogous to those typically employed in Smith-Waterman implementations using an affine gap model. One score matrix, S_{aln}, tracks scores through aligned (matched or mismatched) bases. Two more score matrices, S_{gact} and S_{ghap}, track alignment scores through gaps in the active region (x) or a gaps on the haplotype (y), respectively. The bases of x are positioned along the vertical axis of each matrix, and the bases of y are positioned along the horizontal axis.

*Memoization means that intermediate data is stored instead of being re-computed. The spelling of this term is not a typo.

The first base of x and y are represented in row 1 and column 1, respectively. Each base of the anchor k-mer is added to y, and one column is created in all matrices for each base of y. The initial alignment score, R_{init} is assigned over the anchor k-mer (**Supplementary Eqn. 2**), and all other scores in all three matrices are initialized to 0. A fourth matrix, T, contains traceback information from the end of an alignment to $S_{aln}(0,0)$. A row and a column with index 0 exists with no bases aligned to them. T is initialized so that there is one sub-alignment over the anchor k-mer (**Supplementary Eqn. 3**). This initialization of S_{aln} and T matches each base of the active region and haplotype over the anchor k-mer that seeds haplotype reconstruction, and all acceptable alignments must enter this path at $S_{aln}(k,k)$.

$$\begin{aligned} S_{aln}(i,i) &= R_{init}\,, 0 \leq i \leq k && (2) \\ T(i,i) &\rightarrow T(i-1,i-1), 1 \leq i \leq k && (3) \end{aligned}$$

Storing the whole of all four matrices would result in a large memory footprint, and **Supplementary Section 2.1** outlines how the software implements this algorithm more efficiently.

$$\begin{aligned} S_{aln} &:= \text{Score matrix for alignments through aligned bases.} \\ S_{gact} &:= \text{Score matrix for alignments through insertions (active region gaps).} \\ S_{ghap} &:= \text{Score matrix for alignments through deletions (haplotype gaps).} \\ T &:= \text{The traceback matrix.} \end{aligned}$$

1.6 Alignment score matrices

The score matrices are built using the usual alignment algorithm, but with the Kestrel modifications. For example, transitioning to $S_{aln}(i,j)$ requires adding $match(i,j)$ to each $S_{aln}(i-1,j-1)$, $S_{gact}(i-1,j-1)$, or $S_{ghap}(i-1,j-1)$ that are above 0 and choosing the maximum value. $T(i,j)$ is updated to link $S_{aln}(i,j)$ to the cell or cells that yielded the maximum score. If all scores in $S_{aln}(i-1,j-1)$, $S_{gact}(i-1,j-1)$, and $S_{ghap}(i-1,j-1)$ are 0, or if the computed score is 0 or less, then $S_{aln}(i,j) = 0$ and no link is added to T. **Supplementary Eqn. 4** outlines assignment of $S_{aln}(i,j)$.

$S_{gact}(i,j)$ is set by finding all non-zero scores from $(i,j-1)$ in each score matrix. R_{open} is added to the scores from S_{aln} and S_{ghap}, and R_{gap} is added to all scores. If it is above 0, then the maximum score is recorded in $S_{gact}(i,j)$ and T is updated. **Supplementary Eqn. 5** describes this calculation, and **Supplementary Eqn. 6** describes a similar calculation for S_{ghap}.

$$S_{aln}(i,j) = \max \begin{cases} 0 & \\ S_{aln}(i-1,j-1) + match(i,j) & : S_{aln}(i-1,j-1) > 0 \\ S_{gact}(i-1,j-1) + match(i,j) & : S_{gact}(i-1,j-1) > 0 \\ S_{ghap}(i-1,j-1) + match(i,j) & : S_{ghap}(i-1,j-1) > 0 \end{cases} \quad (4)$$

$$S_{gact}(i,j) = \max \begin{cases} 0 & \\ S_{aln}(i,j-1) + R_{open} + R_{gap} & : S_{aln}(i,j-1) > 0 \\ S_{gact}(i,j-1) + R_{gap} & : S_{gact}(i,j-1) > 0 \\ S_{ghap}(i,j-1) + R_{open} + R_{gap} & : S_{ghap}(i,j-1) > 0 \end{cases} \quad (5)$$

$$S_{ghap}(i,j) = \max \begin{cases} 0 & \\ S_{aln}(i-1,j) + R_{open} + R_{gap} & : S_{aln}(i-1,j) > 0 \\ S_{gact}(i-1,j) + R_{open} + R_{gap} & : S_{gact}(i-1,j) > 0 \\ S_{ghap}(i-1,j) + R_{gap} & : S_{ghap}(i-1,j) > 0 \end{cases} \quad (6)$$

1.7 Alignment extension

Recall from **Supplementary Section 1.3** that the start of the active region, x, was found by locating neighboring k-mers where the frequency difference exceeded a threshold, $N_i > N_{i+1} + \epsilon$. The haplotype, y, is initialized using the k-mer associated with N_i, the left anchor. As described in **Supplementary Section 1.5**, all bases in that anchor initialize the alignment.

The next k-mer after the anchor is altered, and so it has a low frequency. Since the anchor k-mer shares $(k-1)$ bases with the next k-mer, it can be permuted to find what the next base should be. This starts with removing the first base of the anchor to create a $(k-1)$-mer. Then, each possible base is appended to this $(k-1)$-mer, and frequency for each resulting k-mer is retrieved. The base with the maximum frequency is appended to y, and the alignment is updated. The new k-mer is then used to find the next base, and the process repeats until y is fully constructed. Because active region detection takes place using the forward and reverse-complement k-mers, a parallel process on the reverse-complement is performed, and the k-mer frequencies are summed. If no bases produce a k-mer with an acceptable frequency, the alignment is terminated.

If more than one base produces an acceptable k-mer frequency, then one of the bases is saved along with the alignment state, and the saved state is resumed after another alignment completes. Therefore, more than one haplotype, y, may be found for each active region x.

If the active region has no left anchor, and if variant calling without both anchors is enabled, then its right anchor seeds the alignment. In this case, whole alignment process takes place in reverse. Each y is then inverted back to its original configuration to match x.

1.8 Maximum score and optimal alignments

After each base of y is added to the alignment, the overall score can be examined. Since the alignment must cover all of x, only the last row of the alignment score matrix, S_{aln}, needs to be queried. **Supplementary Eqn. 7** defines R_{max}, the maximum alignment score.

$$R_{max} = \max\left(\{S_{aln}(|x|, j),\ 0 \leq j \leq |y|\}\right) \tag{7}$$

If x extends to the end of the reference, X, then it is possible that the alignment ends in a deletion because there is no anchor k-mer on that end. In this case, the maximum score can be computed as the maximum of the final row in both S_{aln} and S_{ghap}. The maximum score is never calculated from the S_{gact} because inserting bases on the end of x can only lower the maximum score. The full alignment is found by traversing the traceback matrix, T, from the cell with the maximum score to $S_{aln}(0, 0)$.

If any cell of T has more than one path out, then there is more than one optimal alignment, and the first alignment as defined by the alignment sort order is used. When comparing two alignments, the one with the first non-matching base comes first. If the non-matching bases agree (same variant), then the next non-matching base is queried. If the non-matching bases do not agree, then alignments are prioritized by mismatch, insertion, and deletion, in that order. This gives the algorithm predictable output for cases such as a deletion in a homopolymer repeat; Kestrel will always report that the first base was deleted even though the alignment score would be the same for a deletion at any locus of the repeat. This effectively left-aligns the variants within the active region.

1.9 Alignment termination

The extension of y must be terminated when the best possible score is reached. Each time a base is added to the alignment, the maximum alignment score is known. However, the maximum possible alignment score that could be obtained by adding more bases to y must also be known. When this maximum potential

score is less than the maximum alignment score, then the alignment cannot be improved by extending y further.

The maximum potential score is determined by examining the last column of S_{aln}. The best possible score that can be obtained from any cell is the case where all subsequent bases of x are aligned with matching bases in y. **Supplementary Eqn. 8** defines $maxpot(i, j)$, the maximum potential score from cell $S_{aln}(i, j)$. **Supplementary Eqn. 9** defines R_{maxpot} as the maximum $maxpot(i, j)$ of the last column of S_{aln}. If $R_{max} > R_{maxpot}$ (See **Supplementary Eqn. 7**), a greater or equal score cannot be obtained by adding more bases to y.

$$maxpot(i,j) = \begin{cases} S_{aln}(i,j) + (|x| - i) \cdot R_{match} & : S_{aln}(i,j) > 0 \\ 0 & : S_{aln}(i,j) = 0 \end{cases} \tag{8}$$

$$R_{maxpot} = \max\left(\{maxpot(i, |y|),\ 0 \le i \le |x|\}\right) \tag{9}$$

When the alignment extends to an end of the reference sequence, then the maximum potential score from the deletion score matrix, S_{ghap}, must also be considered.

The modifications to Smith-Waterman outlined in **Supplementary Section 1.4** are important to make the termination condition deterministic when y does not align well with x because the algorithm gives up on alignments that pass through a cell with a zero score. This is also the reason R_{gap} may not be 0; if it were 0, then the alignment could attach to another region of the genome and extend without bound. These modifications allow degenerate cases to be limited before spending many CPU cycles trying to solve it.

An active region may have more than one haplotype, and a haplotype may have more than one alignment.

1.10 Haplotypes

Each alignment is a haplotype over the active region, and variants are called by tracing the alignment back through matches, mismatches, insertions, and deletions. **Supplementary Section 1.8** outlines how multiple optimal alignments are handled.

Variants called from all haplotypes over the active region are merged so that the same variant is not represented more than once. The depth of the sequence data over the variant is estimated by summing the minimum k-mer count from each haplotype the variant was found in. This allows for a mixing of homozygous and heterozygous events in a diploid organism. In this case, multiple haplotypes would be identified, and although some k-mers are shared among them, the heterozygous k-mers would represent the lowest-count k-mers in each haplotype. Since a homozygous event would be found in all haplotypes, summing the minimum-count k-mer in each haplotype would add to the correct depth. **Supplementary Fig. 6** shows that this method effectively recovers the depth in a monoploid organism even when alignments cannot.

1.11 Parameter selection

The most visible parameter is the k-mer size, k, which should be selected to balance genome complexity with expected error rates. The majority of k-mers must match one region of the genome. When a k-mer maps to multiple regions, the frequencies from both will be mixed together. This will hinder both active region detection and assembly. If the k-mer size approaches the read size, few k-mers will be extracted from each read and the observed coverage will decline. A high error rate in the sequencing data will also cause an observed loss of coverage.

Active regions are detected when the absolute difference between neighboring k-mers exceeds some threshold, ϵ. This parameter is selected by choosing a quantile, Q_ϵ, over the absolute differences of all neighboring

k-mer frequencies. Choosing this parameter depends on how many active regions can be expected, although, choosing a value as high as .95 to .99 works in many cases.

$$Q_\epsilon \quad := \quad \text{A quantile of the frequencies of } N \text{ for choosing } \epsilon.$$

2 Kestrel implementation

This section outlines some of the implementation details in the Kestrel software that make the above algorithm work on real data.

2.1 Alignment matrices

Storing the whole of all four alignment matrices defined in **Supplementary Section 1.5** would require a great deal of memory. This problem would be compounded when multiple haplotypes are searched because all matrices would need to be duplicated. Fortunately, only a small fraction of this data needs to be stored.

Because of the nature of the dynamic programming algorithm, only the last column of the score matrices (S_{aln}, S_{gact}, and S_{ghap}) needs to be stored while the next column is built. Therefore, each of these matrices can be reduced to two arrays where one contains the last column, and one contains the new column as it is added.

Since the active region must be aligned from end to end, the only acceptable alignments contain a non-zero score in the bottom row of the matrix. Since the whole score matrix is not saved, the optimal alignment score must be tracked for each new column, and it is saved separately from the score matrix arrays. When a column is added that has a greater score than the current maximum, it becomes the new maximum.

The traceback matrix, T, is more complex because it is not stored as a matrix. Instead, it is a linked-list of alignment states. Following the links always leads back to $S_{aln}(0, 0)$, which is where all alignments begin. When a non-zero score is added to a score matrix, a link is added from that position in the score matrix to T; the data structure for each element of the score matrix array contains both the numeric score and a link to a node in T. The score matrix arrays can be thought of like a knitting needle that leaves a fabric of linked trace-back nodes as is progresses through the alignment.

The nodes in T must also contain more than one link in the case that there is more than one optimal alignment path. Where the first link traverses back toward $S_{aln}(0, 0)$, the second link points to other nodes at the same level. When this second link is not `NULL`, the alignment splits into multiple paths at that location.

In the case that multiple haplotypes are found, the whole alignment must split. This occurs when multiple possible sequences are explored over an active region (not just alternate alignments of the same sequence). As already noted, only the last column of each score matrix must be stored, and this column contains links into T. Since T is a linked list that is only traversed toward $S_{aln}(0, 0)$, one node of the alignment may have several links into it. Therefore, different haplotypes may link to the same node in T where a split occurred, and no part of T needs to be duplicated or saved other than the nodes already stored in the score matrix array. Both haplotypes will converge at the point where they split, and they continue toward $S_{aln}(0, 0)$ along the same path.

The linked list structure has another more subtle property that Java uses to keep memory usage low. When an alignment path reaches a dead end, the node at the end of the path has no reference to it. This allows the Java Virtual Machine (JVM) garbage collector to detect and remove these nodes. In other words, dead branches of T are automatically pruned. This improves scalabilty by reducing the memory requirements for large active regions where many haplotypes are investigated.

2.2 Active region heuristics

In an ideal scenario, sequence data would cover the sample uniformly at all loci, contain no errors, and k-mers would be long enough so that no k-mer would map to any other region of the genome. This is never observed in practice. Sequence reads contain errors, and they are almost never distributed uniformly. Furthermore, it may be impossible to choose a k-mer size that eliminates all clashes with other loci. For an algorithm that relies on k-mer frequencies, this presents a challenge. These situations must be carefully handled by additional heuristics to avoid errors in the results.

2.2.1 Exponential decay

While scanning for the end of an active region, the active region detector searches for a k-mer frequency that is close to the frequency of the anchor k-mer. When the read depth is not uniform over the active region, this recovery threshold may never be found, and the scan may reach the end of the reference sequence.

To address this problem, an exponential decay function, $f(x)$, is employed to reduce the recovery threshold as the active region extends. $f(0)$ is the anchor k-mer frequency, and it approaches a lower bound, f_{min}, asymptotically. By default, $f_{min} = 0.55 \cdot f(0)$ to avoid ending an active region prematurely on a heterozygous variant. $f(x)$ is defined by scaling and shifting the standard exponential decay function, $h(x)$, as highlighted by **Supplementary Eqn. 10** and **Supplementary Eqn. 11**.

$$
\begin{aligned}
h(x) &= e^{-x\lambda} && (10) \\
f(x) &= (f(0) - f_{min}) \cdot h(x) + f_{min} && (11)
\end{aligned}
$$

$h(x)$ is parameterized by λ, which must be also be set, but Kestrel does not configure this parameter directly because it is difficult to know how to choose a reasonable value. Instead, λ is chosen by a configurable parameter, α, that is defined as the proportion of the decay range, $f(0) - f_{min}$, after k k-mers. This provides a more intuitive way to define how rapidly the recovery threshold is allowed to decline. Choosing λ given α is shown in **Supplementary Eqn. 12**. **Supplementary Fig. 1** illustrates exponential decay with two values of α.

$$
\begin{aligned}
h(k) &= \alpha \\
e^{-k\lambda} &= \alpha \\
-k\lambda &= log(\alpha) \\
\lambda &= \frac{-\log(\alpha)}{k} && (12)
\end{aligned}
$$

At k k-mers, the recovery threshold $f(k) = \alpha \cdot (f(0) - f_{min}) + f_{min}$. In other words, $f(k)$ has declined in its range from $f(0)$ to f_{min} by a factor of α. This is true for all nk such that $f(nk) = \alpha^n \cdot (f(0) - f_{min}) + f_{min}$. **Supplementary Eqn. 13** gives a proof for this claim.

$$
\begin{aligned}
h(x) &= e^{-x\lambda} \\
&= e^{-x\frac{-\log(\alpha)}{k}} \\
&= \left(e^{\log(\alpha)}\right)^{\frac{x}{k}} \\
&= \alpha^{\frac{x}{k}} \\
\square & && (13)
\end{aligned}
$$

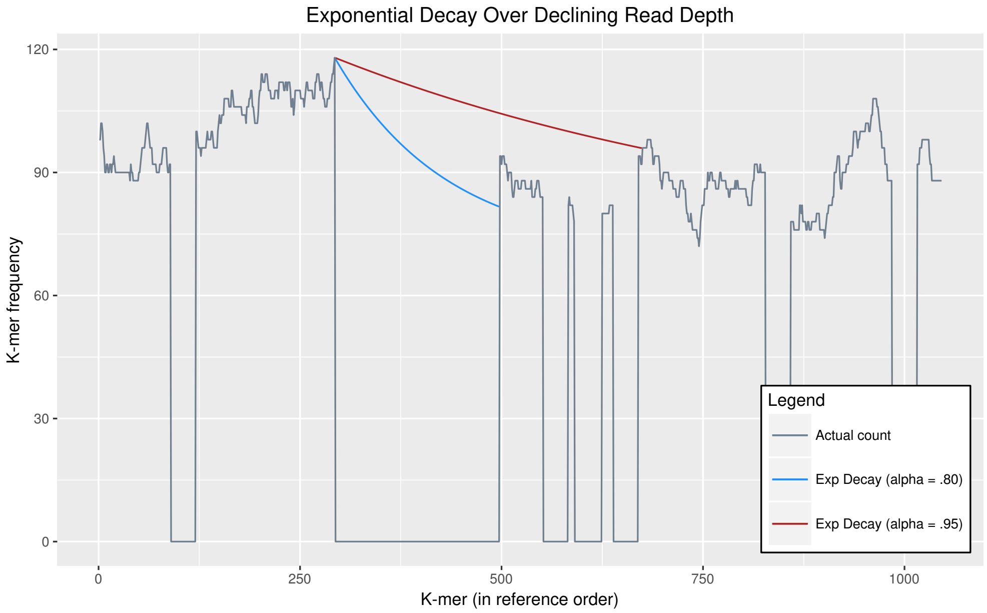

Supplementary Figure 1: To end an active region, the Kestrel algorithm searches for a k-mer frequency that is close to the frequency anchor k-mer. An exponential decay function is applied to the recovery value so that the value declines asymptotically as the scan moves to the right.

$f(x)$:= Exponential decay function for the active region recovery value; $h(x)$ shifted and scaled.

$h(x)$:= The standard exponential decay function, $e^{-x\lambda}$

f_{min} := Lower bound of $f(x)$.

2.2.2 Peak detection

Due to chance or homology, peaks may be observed in the k-mer frequencies of a reference, N. If k is not large enough so that all k-mers map to exactly one locus, then the frequency of some k-mers will be higher because it was found in multiple loci. This causes a peak in the data, and if not properly handled, can lead to an arbitrary active region scan or the premature end of an active region. **Supplementary Fig. 2** shows a peak in an active region where some reference k-mers happened to match another locus. If the active region scan ended on that peak, variants would be missed.

To deal with this problem, Kestrel does not stop immediately when it finds a peak. Instead, it scans ahead to see if the frequency declines again after some number of k-mers (7 k-mers by default). If it does decline within that threshold, then Kestrel passes the peak and continues as if it were not present.

Occasionally, a scan through an active region will encounter many peaks because the scan recovery threshold is at the level of the k-mer frequencies in that region. These peaks are normal fluctuations in the frequencies. When Kestrel finds many peaks, it goes back to the last sharp increase of k-mer counts within the active region scan. If there was no sharp incline, the scan is abandoned and no active region is declared.

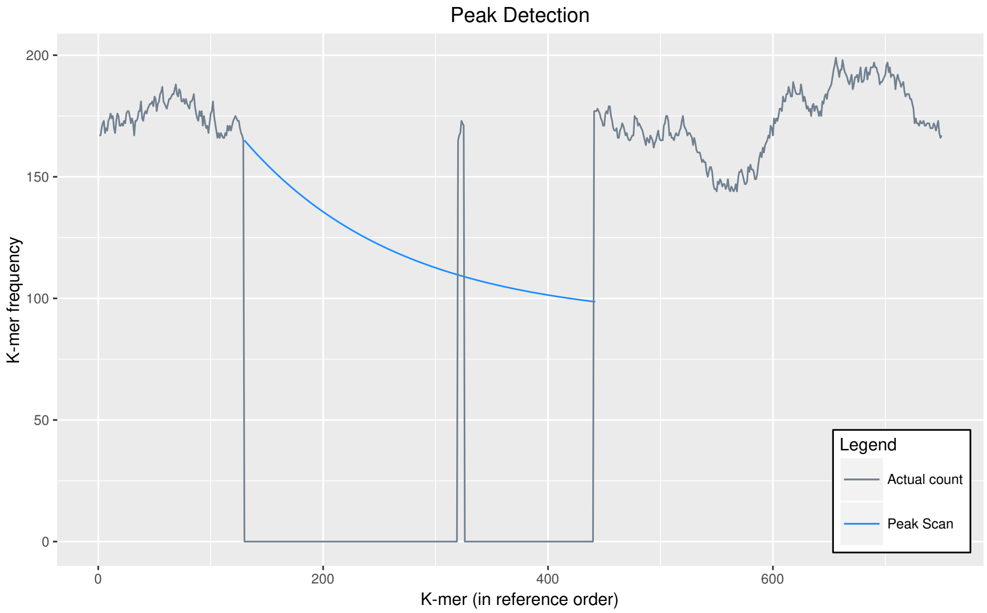

Supplementary Figure 2: Peaks must be detected and bypassed to prevent prematurely ending an active region scan.

3 *S. pneumoniae* test case

3.1 Sequence data

Kestrel was tested using 181 *Streptococcus pneumoniae* (*S. pneumoniae*) whole genome sequencing (WGS) samples released by the Centers for Disease Control and Prevention in NCBI SRA under BioProject PRJNA284954. These data are whole-genome 250 bp paired-end Illumina sequence reads ranging from 14 Mbp to 1,176 Mbp (median = 308 Mbp) and representing 29 distinct serotypes. Four penicillin binding protein (PBP) genes were analyzed (PBP2X, PBP1A, PBP2B, and PBP2A) from a singe reference, TIGR4 (NC_003028.3). The MD5 checksum of this reference sequence, without the FASTA header, whitespace, or newlines, is “50086819690b19b94ce37f1d75a7e539”.

3.2 Variant calling

Variants were called using three approaches. The first is a standard alignment pipeline using BWA[9,10], Picard tools[11], and GATK[4] HaplotypeCaller. The second is Kestrel. The third is a *de novo* assembly pipeline using SPAdes[12], BWA, and SAMtools[13]. All variants identified by the assembly approach were used as the set of true variants to expect from other approaches.

3.2.1 Assembly approach and variant verification

The assembly is used as the standard for measuring the accuracy of GATK and Kestrel. **Supplementary Table 1** shows the steps taken by this approach to find these true variants.

From the assembled scaffolds, only loci that map to the reference with a depth of 1 may be used for verifying variant calls. For any sample, variants called within a region where this is not true cannot be verified and must be ignored for further analysis.

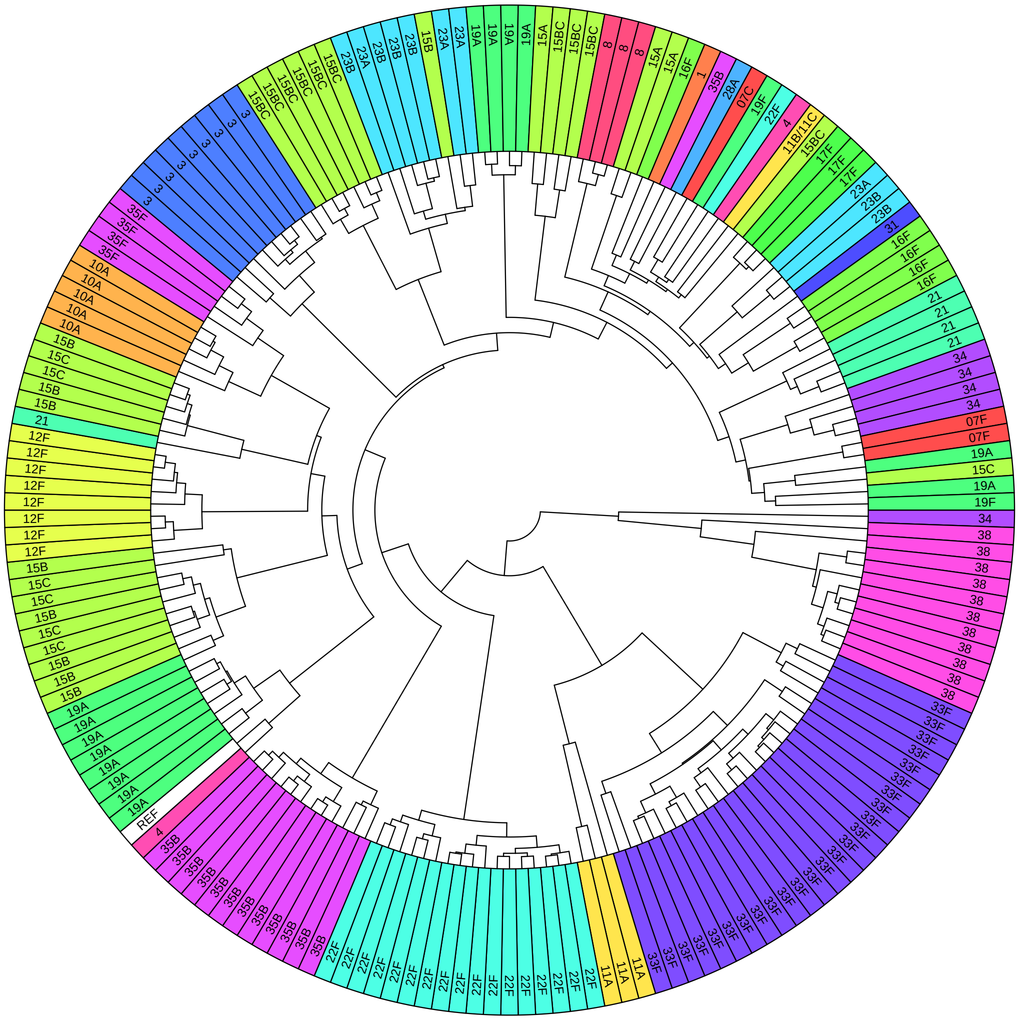

Supplementary Figure 3: Phylogenetic tree showing known serotypes for each sample. Related serotypes are grouped into the same color. The reference sequence, NC_003028.3, is shown in white.

3.2.2 Alignment approach

The alignment approach represents a standard variant-calling pipeline. To remove bias from amplification during sample preparation, duplicates are marked before variant calling. Since mis-alignments often occur around true insertion/deletion (indel) variants, indel realignment is also performed before calling variants. These are the steps recommended by GATK. **Supplementary Table 2** depicts this process.

3.2.3 Kestrel approach

The Kestrel approach represents our novel algorithm and software implementation. Kestrel reads from an indexed k-mer count (IKC) file, and when it is given FASTQ files, it first converts them to an IKC file. We separated these two steps to independently record the time required for each one. **Supplementary Table 3** shows the two steps.

Step	Description	Tool
1	*De novo* assembly of all sequence reads to consensus scaffolds	SPAdes
2	Align all scaffolds to the TIGR4 (NC_003028.3) reference sequence	BWA mem
3	Sort aligned scaffolds	Picard SortSam
4	Generate a pileup of the aligned scaffolds	SAMtools mpileup
5	Call variants from pileup	kescases

Supplementary Table 1: All steps in the assembly approach. The tool "kescases" denotes custom code found in "kescases" repository.

Step	Description	Tool
1	Align all reads to the TIGR4 (NC_003028.3) reference sequence	BWA mem
2	Sort aligned reads	Picard SortSam
3	Mark duplicates	Picard MarkDuplicatesWithMateCigar
4	Indel realignment: Create targets	GATK RealignerTargetCreator
5	Indel realignment: Realign	GATK IndelRealigner
6	Call variants	GATK HaplotypeCaller

Supplementary Table 2: All steps in the alignment approach.

Kestrel uses the KAnalyze application programming interface (API) to interface with IKC files, including when Kestrel converts FASTQ files to IKC files. Although KAnalyze and Kestrel can use k-mers of arbitrary size, we use 31-mers because it appears to work well for most bacterial sequence data. We set a minimum k-mer frequency of 5 to remove sequence-read errors. All variants with a relative coverage (depth of variant / depth of locus) of less than 0.5 were also filtered out by Kestrel.

3.3 Comparing variants

Variants from Kestrel and GATK were evaluated using RTGTools vcfeval[14] with the variants from the assembly as a set of trusted calls. In each sample, only regions with a scaffold alignment depth of 1 were analyzed; variants called by all approaches outside of this region were not used for evaluation and summary statistics.

All true and false calls by Kestrel and GATK are identified as follows:

- **True positive (TP)** A variant that was also called by the assembly approach.
- **False positive (FP)** A variant that was not called by the assembly approach.
- **False negative (FN)** A variant that was not called, but was called by the assembly approach.

From these calls, the sensitivity (true positive rate, or TPR) (**Supplementary Eqn. 14**) and false discovery rate (FDR) (**Supplementary Eqn. 15**) may be calculated. Note that specificity and other statistics that require counting true negatives cannot be calculated because there is no logical way to define true negatives with variant calls.

Step	Description	Tool
1	Generate IKC	KAnalyze
2	Call variants	Kestrel

Supplementary Table 3: All steps in the Kestrel approach.

$$\mathrm{TPR} := \frac{\sum \mathrm{TP}}{\sum \mathrm{TP} + \sum \mathrm{FN}} \quad (14)$$

$$\mathrm{FDR} := \frac{\sum \mathrm{FP}}{\sum \mathrm{TP} + \sum \mathrm{FP}} \quad (15)$$

3.4 Removed samples

During analysis, eight samples and one region of a sample were removed. **Supplementary Table 4** outlines the reasons for removal. Since NC_003028 contains 2,160,842 bp, removing a range from 1 to 2,200,000 effectively removes the sample from analysis. Any samples that were fully removed are not included in figure generation and final results.

chr	accession	start	end	reason
NC_003028	SRR2072251	1,917,888	1,917,912	Declining read coverage likely led to a misassembly
NC_003028	SRR2072306	1	2,200,000	Evidence of contamination in all PBP genes
NC_003028	SRR2072379	1	2,200,000	Evidence of contamination in all PBP genes
NC_003028	SRR2072342	1	2,200,000	Evidence of contamination in all PBP genes
NC_003028	SRR2072298	1	2,200,000	Evidence of contamination in 2X, 1A, and 2B
NC_003028	SRR2072339	1	2,200,000	Evidence of contamination in 2X, 1A, and 2B
NC_003028	SRR2072351	1	2,200,000	Evidence of contamination in all PBP genes
NC_003028	SRR2072219	1	2,200,000	Incomplete sequence coverage
NC_003028	SRR2072360	1	2,200,000	Incomplete sequence coverage

Supplementary Table 4: Regions removed from analysis.

While analyzing the Kestrel variant calls, we noticed particular regions or samples where there was a high concentration of false-negative (FN) calls, and so we evaluated each one to find a cause.

SRR2072251 only showed one 25 bp region with many FNs. After examining the read depth, it appears that coverage dropped significantly to almost 0. The coverage was too small to meet the minimum k-mer count, and we believe this affected the assembly as well. Without a truth-set for comparing variants against and incomplete sequence data for Kestrel, the variants were masked from this region.

In samples SRR2072306, SRR2072379, SRR2072342, SRR2072298, SRR2072339, and SRR2072351, we noticed that there were many false variant calls with a relative depth of 0.70 or less. The IKC files for these samples was also larger than others suggesting that there was more unique sequence in these samples than others. We believe there is some other source of genetic material other than the host genome confounding analysis. This may be contamination or some other genetic material, such as plasmids. Genome assemblies are going to represent a single haplotype, and it may be a mosaic of the alleles present in the sample. Without a way to address these issues using the genome assembly truth-set, we had to remove these samples from our results.

Samples SRR2072219 and SRR2072360 also suffered from a loss of coverage over the PBP genes, which affected the genome assembly and Kestrel calls. The IKC files are also much smaller for these two samples supporting the conclusion that they contain less unique sequence than the whole genome. Because of a systemic loss of coverage affecting the assemblies and variant calling in all pipelines, we removed them from our results.

3.5 Results

The results are reported in the publication text. **Supplementary Fig. 4** is a larger version of the **Publication Fig.2(a)**. The phylogenetic tree shown in **Supplementary Fig. 3** and **Supplementary Fig. 4** were generated using average nucleotide identity (ANI)[15].

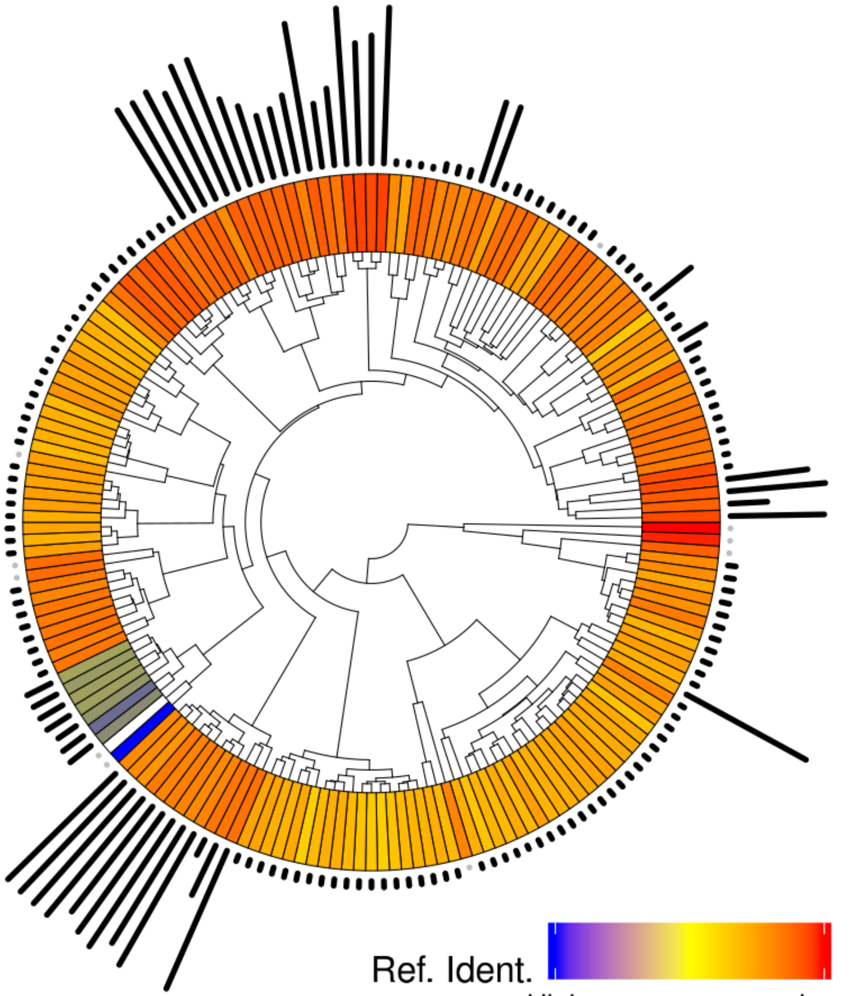

Supplementary Figure 4: Inner track: The phylogeny of all samples by ANI. Middle track: A blue-yellow-red heatmap depicting the distance of each sample from the reference by ANI. Outer track: The relative number of variants identified per sample.

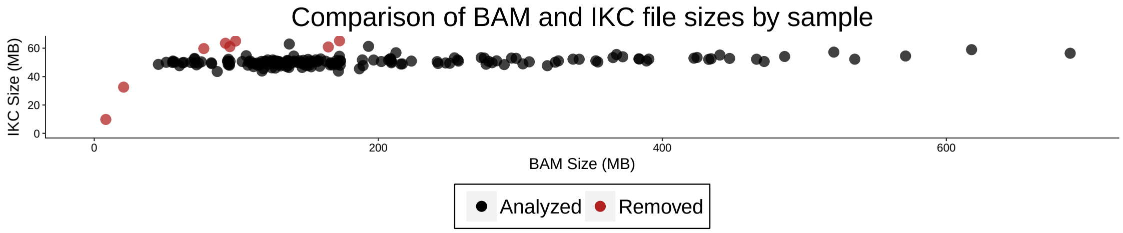

Supplementary Figure 5: The size of IKC files compared with BAM files for each sample. The red samples are those that were removed because there was evidence of contamination or incomplete sequence.

3.6 Data storage

One advantage of IKC files is that they do not grow considerably with read depth. The number of unique k-mers in a sample is fixed, and once the sequencing depth reaches 100% coverage, no new k-mers can come from the original DNA in the sample. Sequencing errors do accumulate, but IKC files for this experiment are filtered with a minimum frequency of 5, and so the majority of these k-mers are removed. BAM files, however, store each read, and so they grow as the read depth accumulates.

Supplementary Fig. 5 compares the size of IKC and BAM files for each sample. The size of IKC files is relatively constant, but the BAM file size is more variable. The 6 removed samples (SRR2072298, SRR2072306, SRR2072339, SRR2072342, SRR2072351, and SRR2072379) are colored red, and these appear to contain more unique k-mers because the IKC file size is larger. Based on this analysis, two more samples (SRR2072345 and SRR2072352) may also include more sequence than the typical sample, but we saw no evidence for this in the variant calls over the PBP genes. Two samples (SRR2072219 and SRR2072360) dropped below the distribution of sizes for IKC files indicating that the whole genome was not captured. Note that the only Kestrel false negative calls were in these two samples.

The size of IKC files was smaller compared to BAM files with averages of 52 Megabytes (MB) and 195 MB respectively. However, since the size of intermediate files needed to generate the IKC is significantly larger with an average of 639 MB, disk savings is only realized when the IKC or BAM file is stored for further analysis.

3.7 Variant Call Depth

Kestrel uses k-mer counts to estimate variant call depth (**Supplementary Fig. 6**). Although k-mers are easily lost to sequencing errors, the reconstructed haplotypes are able to recover a depth estimate even in regions where the alignment depth declines due to poor alignments.

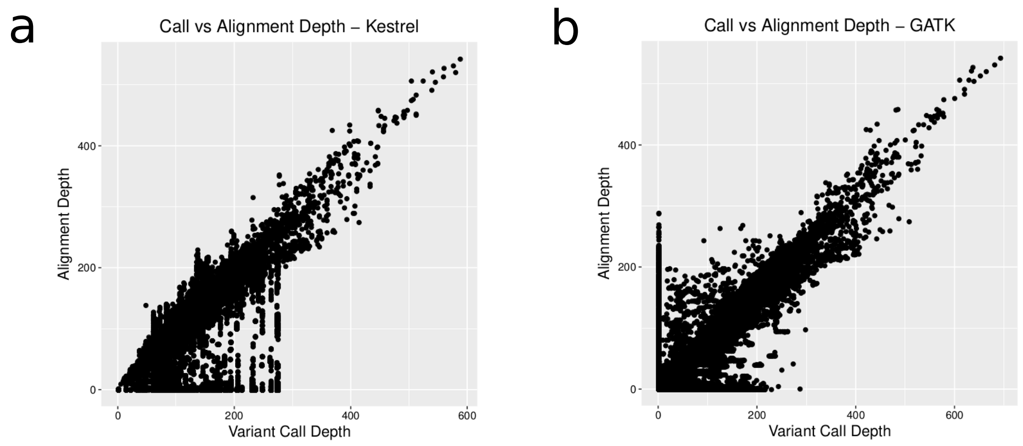

Supplementary Figure 6: Variant depth by the caller (horizontal axis) vs alignment variant depth (vertical axis) for Kestrel (**a**) and GATK (**a**). Kestrel does not show a pattern for underestimating variant depth despite using only k-mers to compute it. Points below the distribution are likely from haplotypes with many variants near a region where alignment depth was lost, but the k-mer counts were able to recover it.

4 *E. coli* test case

309 *Escherichia coli* (*E. coli*) assemblies were obtained[16] and aligned to an *E. coli* K-12 reference (NC_000913.3). Concordant regions where obtained by finding all regions where the alignment depth is exactly 1. Variants were called from the concordant regions of the alignment and used as a set of true variants for benchmarking Kestrel performance.

150 bp paired-end Illumina reads were simulated with ART[17] over all contigs regardless of their alignment. All reads were subjected to k-mer counting, and an IKC file was generated. Variant calling by Kestrel and GATK was performed over the whole genome, and variants in regions where the contig alignment depth was 1 (concordant regions) was used for benchmarking. A second set of variants was analyzed by finding all regions where Kestrel made a local assembly (haplotype regions). All variants were benchmarked by applying vcfeval[14] to variant calls in concordant and haplotype regions and using the contig variant calls from the same regions as a set of true calls.

Although Kestrel had a lower overall sensitivity than the alignment method, there were regions where it performed much better. We found all regions where Kestrel exhibited TP calls that were missed by the alignment method. We took all TP variant calls from both methods and removed all variant calls from Kestrel that were not within 50 bp of a GATK call. Regions within 50 bp were merged into a contiguous region. We found 780 regions ranging from 1 bp (306 regions) to more than 50 bp (35 regions) with a max of size of 114 bp. 86 genes (2% of 4,452 annotated genes) had Kestrel-only calls in at least 20 samples (**Supplementary Fig. 7**, **Supplementary Table 5**).

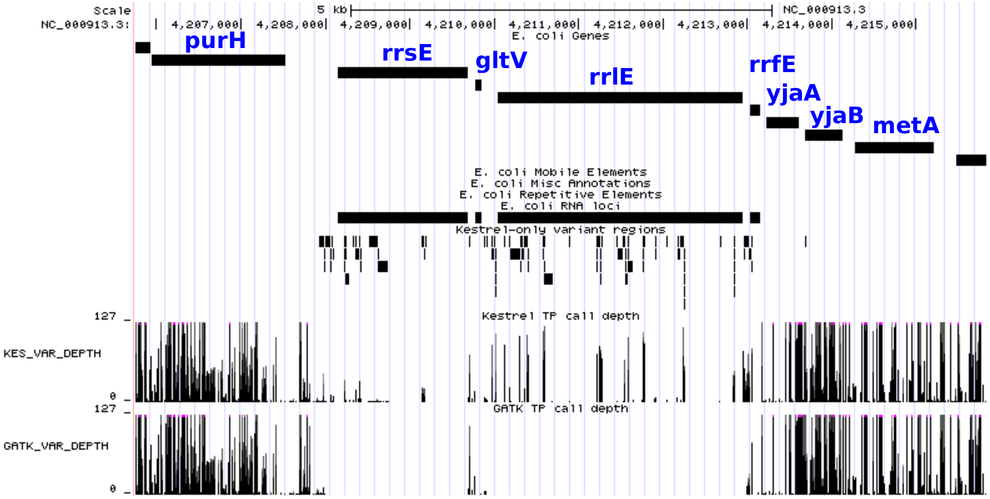

Supplementary Figure 7: An example region of RNA genes where Kestrel made TP calls the alignment method missed. *E. coli* tracks (Genes, Mobile Elements, Misc Annotations, Repetitive Elements, and RNA loci) were extracted from the NCBI genbank file for NC_000913.3. Kestrel-only variant regions were extracted by finding TP Kestrel variants not within 50 bp of TP GATK variants and merging regions within 50 bp of each other. The bottom two tracks show the depth of calls for each approach. Over *rrsE* and *rrlE*, Kestrel reports variant calls which are absent in GATK and are TP by RTG vcfeval.

5 Automated test cases

All analysis was automated, and the code is distributed via the *kescases* package (https://github.com/paudano/kescases).

The software that the analysis pipeline depends on can be built with `make` with rules found in `Makefile`. Two dependencies, `lib/GenomeAnalysisTK.jar` (GATK) and `picard.jar` (Picard Tools), cannot be automatically downloaded and installed, and so they must be obtained separately and copied or linked to the

alx	*bglA*	*crl*	*cspH*	*cvrA*
dapD	*dhaR*	*fadA*	*fliC*	*gadB*
gatR	*gdhA*	*ggt*	*gntP*	*insB1*
insC1	*insD1*	*insE1*	*insF1*	*insH1*
insI1	*insN*	*insO*	*ldrC*	*lomR*
mnmH	*mqo*	*mscS*	*nhaR*	*nohD*
pinQ	*putP*	*qorA*	*renD*	*rhsA*
rhsB	*rrfA*	*rrfB*	*rrfG*	*rrfH*
rrlA	*rrlB*	*rrlC*	*rrlD*	*rrlE*
rrlG	*rrlH*	*rrsA*	*rrsB*	*rrsC*
rrsD	*rrsE*	*rrsH*	*rsxC*	*rzoR*
rzpR	*tfaQ*	*tfaR*	*tfaX*	*topB*
treA	*trmJ*	*ttcA*	*tufA*	*tufB*
yaiT	*yaiX*	*ybeF*	*ybfL*	*ybiU*
ydbA	*ydfB*	*ydfJ*	*yegI*	*yegK*
yghQ	*yhhI*	*yigG*	*yjdF*	*yjeM*
yjeN	*yneK*	*yoeA*	*yqiG*	*yrhA*
gadB				

Supplementary Table 5: Table of *E. coli*genes where at least 1 TP variant was called by Kestrel without a GATK TP call within 50 bp in more than 20 samples.

`lib` directory (see `lib/NOTES`). To build all dependencies once the GATK and Picard Tools libraries are in `lib`, run `make`. To build all freely available dependencies (all except for GATK and Picard), run `make allfree`.

Directory structure overview:

- **Makefile** Master file for building the software packages needed by the analysis process.
- **Snakefile** Master Snakemake file for the test process.
- **bin** Location of executable files generated by `Makefile`.
- **build** Build directories for the software dependencies built by `Makefile`.
- **config** Contains a configuration file used by Snakemake to locate resources.
- **data** Data files used by the build process. Contains mostl references and accessions.
- **kescaseslib** A Python library used by Snakemake.
- **lib** Built libraries are stored in this directory.
- **local** All results, plots, and intermediate data generated by Snakemake. This directory does not appear until the analysis pipeline is executed.
- **rules** Snakemake rules imported by `Snakefile`.
- **scripts** Scripts executed by the Snakemake process. These are mostly R scripts to generate plots.

5.1 *S. pneumoniae*

Directory structure of `local/strep`:

- **ani** ANI input and output. This is used for the phylogeny plots.
- **reference** Reference sequence and indices.
- **results** Raw results and logs.

- **samples** Sample input data.
- **summary** Summary tables and figures.
- **temp** Temporary files that are automatically deleted by the Snakemake process when they are no longer needed.

5.2 *N. meningitidis*

Directory structure of `local/mlst`:

- **results** Raw results and logs.
- **samples** Sample input data.
- **summary** Summary tables and figures.

5.3 *E. coli*

Directory structure of `local/ecoli`:

- **reference** Reference sequence and indices.
- **results** Raw results and logs.
- **summary** Summary tables and figures.
- **temp** Temporary files that are automatically deleted by the Snakemake process when they are no longer needed.

References

[1] Gardner, S. N. & Slezak, T. Scalable SNP Analyses of 100+ Bacterial or Viral Genomes. *Journal of Forensic Research* **1**, 107 (2010).

[2] Gardner, S. N. & Hall, B. G. When Whole-Genome Alignments Just Won't Work: kSNP v2 Software for Alignment-Free SNP Discovery and Phylogenetics of Hundreds of Microbial Genomes. *PloS One* **8**, e81760 (2013).

[3] Audano, P. & Vannberg, F. KAnalyze: a fast versatile pipelined K-mer toolkit. *Bioinformatics* **30**, 2070–2 (2014).

[4] McKenna, A. *et al.* The Genome Analysis Toolkit: A MapReduce framework for analyzing next-generation DNA sequencing data. *Genome Research* **20**, 1297–1303 (2010).

[5] Marçais, G. & Kingsford, C. A fast, lock-free approach for efficient parallel counting of occurrences of k-mers. *Bioinformatics* **27**, 764–770 (2011).

[6] Rizk, G., Lavenier, D. & Chikhi, R. DSK: k-mer counting with very low memory usage. *Bioinformatics* **29**, 652–653 (2013).

[7] Smith, T. & Waterman, M. Identification of common molecular subsequences. *Journal of Molecular Biology* **147**, 195–197 (1981).

[8] Eddy, S. R. What is dynamic programming? *Nature Biotechnology* **22**, 909–910 (2004).

[9] Li, H. & Durbin, R. Fast and accurate short read alignment with Burrows-Wheeler transform. *Bioinformatics* **25**, 1754–1760 (2009).

[10] Li, H. & Durbin, R. Fast and accurate long-read alignment with Burrows-Wheeler transform. *Bioinformatics* **26**, 589–595 (2010).

[11] Institute, B. Picard tools (2016). URL http://broadinstitute.github.io/picard/. Accessed April 22, 2016.

[12] Bankevich, A. *et al.* SPAdes: A New Genome Assembly Algorithm and Its Applications to Single-Cell Sequencing. *Journal of Computational Biology* **19**, 455–477 (2012).

[13] Li, H. *et al.* The Sequence Alignment/Map format and SAMtools. *Bioinformatics* **25**, 2078–2079 (2009).

[14] Cleary, J. G. *et al.* Comparing Variant Call Files for Performance Benchmarking of Next-Generation Sequencing Variant Calling Pipelines. *bioRxiv* 023754 (2015).

[15] Goris, J. *et al.* DNA-DNA hybridization values and their relationship to whole-genome sequence similarities. *International Journal of Systematic and Evolutionary Microbiology* **57**, 81–91 (2007).

[16] Salipante, S. J. *et al.* Large-scale genomic sequencing of extraintestinal pathogenic Escherichia coli strains 1–10 (2015).

[17] Huang, W., Li, L., Myers, J. R. & Marth, G. T. ART: A next-generation sequencing read simulator. *Bioinformatics* **28**, 593–594 (2012).
